# When Combatting Ebola and other Outbreak-Prone Pathogens, Global Health is American’s Health

**DOI:** 10.4269/ajtmh.25-0153

**Published:** 2025-03-13

**Authors:** Daniel G. Bausch, David Heymann

**Affiliations:** ^1^ American Society of Tropical Medicine and Hygiene, Arlington, Virginia, USA;; ^2^ Center for Infectious Disease Emergency Response, Yong Loo Lin School of Medicine, National University of Singapore;; ^3^ London School of Hygiene and Tropical Medicine, London, United Kingdom

On January 30, 2025, an outbreak of Ebola virus disease (EVD) was declared in Uganda, caused by the Sudan ebolavirus species.^1^ This virus, found in Central Africa, has caused death in up to 70% of cases of some outbreaks.^2^ Controlling such dangerous outbreaks requires a coordinated global response to stop transmission where the viruses first emerge in Africa, not only to limit suffering there, but also to prevent amplification into full-blown epidemics that threaten neighboring as well as distant countries, including the United States.

Although most EVD outbreaks have been small, the 2013–2016 outbreak in West Africa led to 28,646 cases and 11,323 deaths—numbers considered by most experts to be drastic underestimates.^3^^,^^4^ Although cases were largely confined to West Africa, the outbreak caused global economic and political upheaval, demonstrating the potential of infectious disease outbreaks in far-away places to snowball from a local catastrophe into a devastating international crisis, with potential implications for United States national security.^5^

Factoring in impacts on health workers, long-term health conditions suffered by 17,000 EVD survivors, and the costs of treatment, infection control, screening, and deployment of personnel beyond West Africa, the 2013–2016 EVD epidemic cost an estimated $53 billion, globally.^6^ This included, in the United States, a cost of more than $2 billion and the loss of over 10,000 jobs tied to exports.^7^ The United States contributed extensive funds, including $5.4 billion directly to response efforts in West Africa, $1.5 billion to the World Health Organization (WHO) to support regional response, and undoubtedly billions more to establish domestic surveillance of returning travelers, advance urgently needed scientific research on prevention and treatment of EVD, and foster health and economic recovery in the afflicted countries post-outbreak.^8^^,^^9^

United States Government experts played a major role in the 2013–2016 EVD outbreak response in West Africa. The U.S. Centers for Disease Control and Prevention (CDC) deployed over one thousand experts, who made major contributions to case finding and contact tracing, laboratory diagnosis, and infection prevention and control.^7^^,^^10^ U.S. National Institutes of Health (NIH) scientists conducted research at the NIH intramural laboratories and collaborated with African researchers and the WHO on clinical trials in West Africa to develop vaccines and treatments, as well as on field studies to better understand how Ebola virus was transmitted—all to enable improvement of control measures.^11^^–^^13^ The U.S. Agency for International Development worked in partnership with host governments, international donors, and partners to assist afflicted countries in West Africa with recoveries in health, education, agriculture and food security, governance, technology, and innovation.^14^ The U.S. Military deployed over 3,000 personnel in wide-ranging support for clinical care, diagnostics, and logistics.^15^^,^^16^ Meanwhile, at home in the United States, a host of federal and state workers established surveillance systems to rapidly detect imported cases and provide the safest state-of-the-art medical care when they occurred.^17^^,^^18^

Despite the EVD outbreak’s terrible toll in West Africa, most experts believe that it could have been much worse.^3^^,^^4^ In terms of absolute cases and deaths, the impact outside Africa was small; excluding 20 planned and controlled medical evacuations, there were seven cases imported to countries outside of Africa, including a total of two to the United States—one fatal, with two subsequent non-fatal secondary infections.^4^

Given so few cases in the United States, should we conclude that the billions spent were wasted? Leaving aside moral arguments, made by many compassionate American citizens, about a duty to alleviate human suffering wherever it may occur, one might conclude that outbreaks of rare viruses overseas are simply “not America’s problem.” However, it is unlikely that success in controlling the EVD outbreak in Africa and limiting its direct impact to the United States was due only to luck. Rather, this control was almost certainly the result of the collective efforts and investments of international organizations, governments, healthcare workers, and other public health entities around the world, and in particular in the United States.

In the decade since the major epidemic in West Africa, EVD outbreaks have occurred with increasing frequency, with eleven since 2015.^19^ But, with the exception of a major outbreak (3,470 cases with 2,280 deaths) in eastern Democratic Republic of the Congo in 2018–2020, in which control was severely hampered by civil unrest in the region, all of these outbreaks have been rapidly contained;^20^ a 2022 outbreak in Uganda of 164 cases was the largest, with no cases exported outside Africa. Again, this promising track record over the last decade was not due only to luck, but rather the result of international technical collaboration and investment, including from the United States. CDC has consistently provided fundamental support to colleagues in Africa, and NIH spearheaded a clinical trial during the 2018–2020 outbreak that, for the first time, demonstrated two drugs effective against EVD.^21^^–^^23^ Meanwhile, WHO has been instrumental in coordinating large clinical trials of EVD vaccines, which have led to two licensed vaccines for Zaire ebolavirus that have been instrumental in protecting entire communities from the deaths, suffering, and upheaval caused by this dangerous disease.^24^^,^^25^ With WHO support, a trial of a vaccine for Sudan ebolavirus has recently begun in Uganda.^26^

Recent policies of the new United States administration, including withdrawal from the WHO,^27^ defunding and dismantling the U.S. Agency for International Development,^28^ destabilizing federal health and research programs such as the NIH and Indian Health Service through massive job terminations,^29^ and limiting communication between CDC and WHO,^30^ not only severely undermine the United States leadership in and contribution to global health, but also undermine our national security. While we withdraw, Ebola advances. And the list does not stop with Ebola—Marburg virus, mpox, avian influenza, coronaviruses—there is no shortage of microbial threats in our ever-interconnected world. Without United States involvement, both financial and technical, the world be greatly hindered in responding to such dangers, and risks to the United States will grow.

Effective global health interventions are often a victim of their own success. The general public may not notice the threat of a disease that does not come to their doorstep or appreciate the outbreak that does not happen. While few would oppose efforts to improve the efficiency of United States government operations, make no mistake— keeping the United States and the world safe does not happen passively. If we are not threatened by an outbreak today or if an outbreak that did occur was not more severe, it was very likely because of our significant investment in outbreak control.

When it comes to global health, international engagement, including financial and technical support and collaboration between scientists and public health experts around the globe, is essential. In today’s modern and mobile world, diseases know no boundaries. No matter what corner of the planet you live in or whether you care or not what happens with EVD in Uganda right now, it is still in your best interest to support the people and organizations that do.

Global health is America’s health.
